# Is the kidney disease quality of life-36 (KDQOL-36) a valid instrument for Chinese dialysis patients?

**DOI:** 10.1186/1471-2369-15-199

**Published:** 2014-12-15

**Authors:** Susan Ka Yee Chow, Bonnie Mee Ling Tam

**Affiliations:** School of Nursing, The Hong Kong Polytechnic University, Hunghom, Kowloon, Hong Kong

**Keywords:** Chinese, Dialysis patients, Kidney disease quality of life, Reliability, Validity

## Abstract

**Background:**

The aim of this study is to determine the validity and reliability of the Cantonese Chinese version of the Kidney Disease Quality of Life-36 (KDQOL-36™) questionnaire. The scale has been translated into Cantonese Chinese, but has not been tested among the Cantonese-speaking populations.

**Methods:**

A total of 110 dialysis patients and 122 renal transplant patients were recruited. The data for the KDQOL-36™ were extracted from the KDQOL-Short Form. The criterion validity and scale equivalence were examined using the KDQOL-Short Form scores as the gold standard. The Hospital Anxiety and Depression scale was used to identify the correlations between depression, anxiety, and quality of life to establish the convergent validity. Discriminant validity was examined using the transplant patients to compare the quality of life of dialysis patients. The Cronbach’s alpha coefficient and test-retest were used for estimating reliability.

**Results:**

There were very strong positive correlations for the physical and mental component summary between the KDQOL-36™ and KDQOL-Short Form. Despite the strong correlations, the effect size was 0.6 and 0.13 for the physical composite summary and mental composite summary score, respectively. Most of the subscales demonstrated significant moderate correlations with the Hospital Anxiety and Depression Scale, from -0.265 to -0.516. The discriminant validity was confirmed with a significant difference between the dialysis and transplant group patients. A high intraclass correlation of >0.98 was demonstrated in the test-retest.

**Conclusion:**

The Cantonese Chinese KDQOL-36™ was reliable. Further testing will be required to determine its validity for the physical health summary scale.

## Background

According to the World Health Organization, the number of patients who have End Stage Renal Disease (ESRD) and are receiving renal replacement therapy (RRT) is increasing dramatically worldwide. It is estimated that more than 1.4 million people receive RRT, and the incidence of ESRD is growing by around 8% annually [1]. The Hong Kong Renal Registry Report showed that in 2011, 8199 patients received RRT, of which 3401 underwent peritoneal dialysis, 945 received haemodialysis, and 3234 were living with a functioning renal transplant [2]. The above patients were treated by hospitals or dialysis centres operated by the Hospital Authority of Hong Kong. Patients who were receiving RRT solely in the private sector were not included in the report.

The goal in providing RRT is not only to prolong life and maintain health but also to sustain the patient’s quality of life (QoL) [3]. Given the potentially profound physical changes resulting from dialysis, clinicians need to monitor patients not only for physical health but for psychological health. In the United States, the Center for Medicare Services now requires dialysis facilities to perform routine measurements of Health Related Quality of Life, preferably using the Kidney Disease Quality of Life-36 questionnaire (KDQOL-36™), with additional instruments if appropriate [4].

The Kidney Disease Quality of Life (KDQOL™) is a self-reported questionnaire that combines the generic SF-36 Health Survey instrument and disease-specific components for assessing the health-related quality of life of chronic kidney disease patients [5]. A short version of the KDQOL-SF™, which consists of eight subscales with 43 items on kidney disease and SF-36, was developed by the same author. An even briefer version is becoming popular in clinical trials because it can be completed in a short time. This is the KDQOL-36™, an abbreviated form of the KDQOL-SF™, which consists of the SF-12 Health Survey instrument plus 24 items on quality of life related to kidney disease [6]. The Cantonese Chinese version of the scale was translated by Amgen, Inc. and the MAPI Institute and can be downloaded from the RAND Corporation’s website (http://www.rand.org/health/surveys_tools/kdqol.html) for non-commercial use. The scale has been translated into different Chinese languages, including Cantonese and Mandarin Chinese. Mandarin is the official language of China, while Cantonese is a Chinese dialect. Cantonese is spoken by the majority of people in Hong Kong, Macau, and Guangdong province. The dialect is also spoken by overseas Chinese communities in Southeast Asia, Australia, the United States, and Canada [7]. A proper translation does not guarantee that the scale is reliable and valid in the population. The translated version of the scale has not been evaluated by RAND, and its psychometric properties have not been confirmed in a representative sample of the population. The aim of this study was to determine the validity and reliability of the Cantonese Chinese KDQOL-36™ among Cantonese-speaking Hong Kong Chinese patients undergoing dialysis. The findings can contribute to valuable clinical applications and provide an international comparison of the quality of life among the Cantonese-speaking population.

## Methods

### Structure of the KDQOL-36™

The KDQOL-36™ combines generic domains with disease-specific domains. The disease-specific core has 24 items comprising three scales: Symptoms and Problems (12 items), Burden of Kidney Disease (4 items), and Effects of Kidney Disease (8 items). The items of the three subscales are embedded in the KDQOL-SF™ and they are exactly the same as the KDQOL-SF™. The generic core is the 12-item Short Form Health Survey (SF-12) [8]. The results of the SF-12 instrument are summarized into the Physical Component Summary (PCS) score and the Mental Component Summary (MCS) score. The raw scores are transformed linearly to a range of 0 to 100, with higher scores indicating better HRQOL [5]. The scale was translated by RAND Corporation according to the basic guidelines and specifications on using forward and back translation (see: http://www.rand.org/health/surveys_tools/about_translations.html).

### Sampling and setting

The study protocol was approved by the Human Subjects Ethics Application Review System of the University and the Cluster Research Ethics Committee, Hospital Authority, Hong Kong Special Administrative Region with which the authors were affiliated, and performed in accordance with the ethical standards that had been laid down. All of the subjects gave their written consent prior to their inclusion in the study. They were patients attending the renal dialysis unit of a regional hospital and its satellite dialysis centre in Hong Kong. Two groups of subjects were recruited in this study. The first group consisted of patients over the age of 18 who had been undergoing dialysis treatment for at least three months and who were able to respond to the questionnaire. The second group were patients who had undergone a renal transplant at least one year ago. The criteria for exclusion were patients who had been diagnosed with mental illness and who were not able to respond to the questionnaire. Since the items of KDQOL-36 are embedded in KDQOL-SF, the two groups of patients were required to complete KDQOL-SF and the demographic questionnaire. We retrieved the items of KDQOL-36 from KDQOL-SF for analysis.

With regard to sample size, a good reliability estimate should involve at least 50 or more subjects [9]. The minimum sample size required for testing the validity and reliability of an instrument is 80 and 20 subjects, respectively [10]. In our present study, we set out to compare the QoL of dialysis patients and renal transplant patients. Based on a medium effect size of 0.5 between the two groups, with an alpha of 0.05 and a power of 0.8, the required sample for each group was 64. Based on the above information, the estimated sample size for the study would be no less than 80. A total of 110 dialysis patients and 122 renal transplant patients were included in this study through convenience sampling.

### Validity estimate

A test of an instrument’s validity is an examination of whether the instrument measures what it is supposed to measure, and a variety of approaches should be used rather than a single approach [11]. In this study, the criterion, convergent, and discriminant validity were evaluated.

Since the three subscales for kidney diseases are exactly the same for the KDQOL-SF™ and KDQOL-36™, our study mainly focused on determining the validity of the SF-12 Health Survey, which is embedded in the scale. The validity and reliability of the Chinese Hong Kong version of SF-36 was determined in 1998 [12], and the physical and mental health summary scales were considered valid and reliable in a Chinese population in Hong Kong [13]. The validity of the Chinese version of SF-12 was determined by various authors using data collected from adolescents, healthy adults, and participants with chronic diseases [14–16]. Despite the previous validation, there is still a lack of information on the validity of the Cantonese Chinese of SF-12 for chronic kidney disease patients.

Criterion validity and equivalence is considered critical as it helps to provide evidence of the extent to which the outcomes of a new scale correlate with the outcomes on a criterion test [17]. Since the KDQOL-36™ data were extracted from the KDQOL-SF™, the criterion validity and scale equivalence of the KDQOL-36™ were examined using KDQOL-SF™ scores as the gold standard. The effect size for the PCS and MCS between the KDQOL-SF™ and KDQOL-36™ were used to examine whether the two scales are equivalent. We hypothesized that there should be strong correlations between the KDQOL-SF™ and KDQOL-36™ scores, while the effect size of the two scales would be small. Convergent validity involves investigating the correlational evidence of a measurement using another measure scale [11]. Previous studies showed a positive association between health-related QoL and depression among chronic disease patients, for example, Parkinson’s disease, epilepsy, and chronic dialysis patient groups [18–20]. The Hospital Anxiety and Depression scale was used to identify the correlations between depression and QoL. The lower scores indicate less anxiety and depression. We hypothesized that there would be moderate, negative significant correlations between the outcomes of the two scales. As for discriminant validity, it is an approach to assess the degree to which an instrument yields different results when measuring two different subgroups [21]. The previous studies showed that the transplant group experienced less pain and discomfort, higher energy levels, good mobility, and enjoyed better personal relationships than the dialysis patients [22]. We hypothesized that there would be a significant difference in QoL between the dialysis and transplant group patients.

### Reliability estimate

The reliability of a scale is defined as the ability of an instrument to produce similar results after being repeatedly applied to the same group of subjects [23]. The Cantonese Chinese KDQOL-36™ was administered twice to 20 dialysis patients within an interval of 10 to 14 days to determine the reproducibility of the instrument. Other than test-retest reliability, the Cronbach’s alpha coefficient was examined on the subscales for internal consistency.

### Data analysis

SPSS, version 21.0 (IBM SPSS Inc., Chicago, IL, USA), was used to perform the data analysis. Descriptive statistics were used to examine the demographic characteristics of the participants. The SF-12 data were extracted from the SF-36 data. For validity testing, criterion validity was assessed using Spearman’s rho correlation coefficient between the subscales scores of the KDQOL-SF™ and KDQOL-36™. A further examination was carried out using the effect size to determine whether the KDQOL-36™ gave similar results from those of the KDQOL-SF™. For discriminant validity, an independent t-test was used to compare the QoL of dialysis patients and patients who had undergone a renal transplant. For convergent validity, Spearman Rho correlations were used to examine the strength of the relationship of the KDQOL-36™ with the Hospital Anxiety and Depression scores. The test-retest reliability was estimated using intraclass correlation coefficients (ICC). Internal consistency reliability was evaluated using the Cronbach’s alpha coefficient calculated separately for each subscale. Statistically significant levels were set at a p-value of <0.05.

## Results

### Sample characteristics

The mean age of the dialysis patients was 58.21 ± 15.22 years, while that of the transplant patients was 51.83 ± 10.31 years. The majority of the subjects were married and there were more peritoneal dialysis patients in the dialysis group. With regard to education levels, more than one third of the dialysis patients had a primary school education or less, while 64.7% of the transplant patients had completed secondary school education or above. There were significant differences between the two groups in gender, age and level of education. Please see Table 1 for details.

**Table 1:** **Patient characteristics**

Characteristics	Dialysis group (n = 110)	Transplant group (n = 122)	p-value
Gender	n (%)	n (%)	0.052^c^
Male	75 (68.2%)	68 (55.7%)	
Female	35 (31.8%)	54 (44.3%)	
Age (Mean ± SD)	58.21 ± 15.22	51.83 ± 10.31	*< 0.001^t^
Marital Status, n (%)			0.513 ^c^
Not married	20 (17.3%)	27 (22.3%)	
Married	91 (82.7%)	94 (77.7%)	
Kind of dialysis			
Haemodialysis	36 (32.7%)		
Peritoneal dialysis	74 (67.3%)		
Education			*< 0.001^c^
Primary school or below	40 (36.4%)	12 (10.1%)	
Secondary school but not graduated	29 (26.4%)	30 (25.2%)	
Secondary school	26 (23.6%)	47 (39.5%)	
College or above	15 (13.6%)	30 (25.2%)	

^t^Continuous variables were analyzed by independent-samples t test.

^c^Categorical variables were analyzed by Pearson Chi-square test.

*p < 0.05.

### Validity tests

With regard to criterion validity, there were very strong positive correlations between the KDQOL-36™ and KDQOL-SF™ for the PCS and MCS scores. The correlations between the corresponding summary scores were greater than 0.85 with p < 0.001. With regard to the effect size, it was calculated by dividing this difference by the standard deviation (SD) of the SF-36 summary score [24]. The mean and standard deviation of the PCS for the KDQOL-SF™ and KDQOL-36™ was 36.27 ± 8.31 and 27.44 ± 12.53, respectively, with an effect size of 0.70. For the MCS, the mean and standard deviation for the KDQOL-SF and KDQOL-36 was 41.12 ± 11.27 and 42.92 ± 13.01 and, respectively, with an effect size of 0.14. There were significant differences in PCS and MCS scores among the two scales. Table 2 shows the details.

**Table 2:** **Comparisons of the KDQOL-SF** **™** **and KDQOL-36** **™**

PCS	Mean ± SD
KDQOL-SF™	36.27 ± 8.31
KDQOL-36™	27.44 ± 12.53
Difference	8.82 (p < 0.001)
Correlation	0.847 (p < 0.001)
Effect size	0.70
MCS	
KDQOL-SF™	41.12 ± 11.27
KDQOL-36™	42.92 ± 13.01
Difference	-1.80 (p = 0.003)
Correlation	0.882 (p < 0.001)
Effect size	0.14

The convergent validity was established by exploring the correlations of the subscales with the domains of the Hospital and Anxiety Scale. There were negative low to moderate correlations between anxiety and the PCS and MCS, with *r* = -0.328 (p < 0.001) and *r* = -0.459 (p < 0.001), respectively. The low to moderately negative correlations were found between depression and the PCS and MCS, with *r* = -0.265 (p < 0.05) and *r* = -0.516 (p < 0.001), respectively. The correlations between anxiety, depression, and the kidney disease targeted scales were moderate. The details are given in Table 3. The correlation matrix among the five subscale scores of KDQOL-36™ was shown in Table 4. There were moderate correlations between most of the subscales, except the correlations between PCS and Effects of Kidney Disease, and PCS and MCS scores.

**Table 3:** **Correlations between the KDQOL-36** **™** **and Hospital Anxiety and Depression Scale**

Subscale scores	HADS (Anxiety)	HADS (Depression)
Symptom/problem list	r = -0.49**	r = -0.372**
Effects of kidney disease	r = -0.496**	r = -0.24*
Burden of kidney disease	r = -0.445**	r = -0.396**
KDQOL-36™ (PCS score)	r = -0.247*	r = -0.447**
KDQOL-36™ (MCS score)	r = -0.484**	r = -0.5**

Spearman Rho correlation coefficient, *p < 0.05, **p < 0.001.

**Table 4:** **Correlation coefficients matrix among five subscales scores of KDQOL-36** **™**

	Symptom/problem list	Effects of kidney disease	Burden of kidney disease	KDQOL-36™ (PCS)	KDQOL-36™ (MCS)
Symptom/problem list	1.00				
Effects of kidney disease	0.525**	1.00			
Burden of kidney disease	0.469**	0.371**	1.00		
KDQOL-36™ (PCS)	0.491**	0.184	0.253*	1.00	
KDQOL-36™ (MCS)	0.412**	0.405**	0.347**	0.055	1.00

Spearman Rho correlation coefficient, *p < 0.05, **p < 0.001.

The independent t-test was used to compare the QoL of the dialysis patients and patients who had undergone a renal transplant. There were significant differences between the two groups in the three subscales for kidney disease, and the PCS and MCS of the KDQOL-36. Please refer to Table 5 for details.

**Table 5:** **Comparisons of the QOL of dialysis and transplant patients**

Kidney disease-targeted scales	Dialysis group (n = 110)	Transplant group (n = 122)	p-value
Symptom/problem list	68.75 (58.33 – 82.81)	83.33 (75 – 91.67)	**< 0.001
Effects of kidney disease	56.25 (42.19 – 68.75)	81.25 (65.63 – 90.63)	**< 0.001
Burden of kidney disease	25 (7.81 – 43.75)	62.5 (43.75 – 81.25)	**< 0.001
KDQOL-36™ (PCS)	36.02 (29.94 – 43.36)	47.25 (39.45 – 52.57)	**< 0.001
KDQOL-36™ (MCS)	39.13 (32.49 – 49.16)	49.18 (43.93 – 55.67)	**< 0.001

Mann–Whitney U test **p < 0.05.

### Reliability estimate

With regard to internal consistency, the coefficients of the three subscales related to kidney disease ranged from 0.65 to 0.83, which was evidence of adequate to good internal consistency. The coefficient for the PCS and MCS was 0.32 and 0.53, respectively. Regarding test-retest reliability, the ICCs were above 0.98 for the five subscales. Other than ICCs, the Kappa and Weighted Kappa index were examined. The Kappa index of the five subscales ranged from 0.68-1, whilst the Weighted Kappa index ranged from 0.73-1. The results of the reliability tests are given in Table 6.

**Table 6:** **Internal consistency, test-retest reliability of the PCS, MCS, and kidney disease targeted scales**

Items	Test-retest reliability ICC (Confident interval)	Kappa	Weighted kappa	Cronbach’s alpha (Dialysis group)
Symptom/problem	0.997 (0.991-0.999)	0.826-1	0.851-1	0.833
Effects of kidney disease	0.983 (0.951-0.994)	0.75-1	0.828-1	0.794
Burden of kidney disease	1	1-1	1-1	0.651
PCS	0.993 (0.981-0.998)	0.688-1	0.737-1	0.322
MCS	0.996 (0.989-0.999)			0.537

## Discussion

In summary, the majority of previous studies assessing the validity and reliability of the KDQOL-36™ have been conducted in the West [6, 25]. This is the first time that the Cantonese Chinese KDQOL-36™ has been validated on dialysis patients in Hong Kong. Our results suggest that the scale is reliable and has an acceptable level of validity for understanding the health-related QoL of dialysis patients.

### Criterion validity of the Cantonese Chinese KDQOL-36

There were high correlations, at *r* >0.84, between the physical and mental summary scores of SF-36 and SF-12. SF-12 demonstrated evidence of criterion validity, as the two scales were very similar. Despite the high correlations on the PCS and MCS, our study was unable to draw any conclusions on the equivalence of SF-36 and SF-12 due to the medium effect size of the PCS from the two scales. According to Lam [26], the generally accepted minimal clinically important difference (MCID) standard is 0.5. For those scores with a difference in effect, <0.5 was considered as having measurement equivalence [27]. The moderate effect size was related to the number of items selected for PCS in SF-12 might indicate the loss of crucial information in the short version. In 2005, a Chinese Hong Kong (HK) specific SF-12 was developed where six of the items that were selected were different from those of the standard SF-12 [16]. The scale had a different scoring algorithm and was found to be more sensitive to the Hong Kong Chinese population.

To further investigate the issue of scale equivalence, comparisons were made using the Chinese (HK) specific SF-12 data extracted from the SF-36 data. The results also demonstrated high correlations between SF-36 and the Chinese (HK) specific SF-12 on the PCS and MCS score. Most importantly, the effect size for the PCS was reduced to 0.32, which is within the generally accepted MCID, while the effect size for the MCS was 0.04. Table 7 gives details of the comparisons. The results corroborated Lam’s study that the effect size decreased if the HK specific version was used instead of the standard version for heart disease patients. The difference in effect size could be due to the selected items in the Standard SF-12 versions being not sensitive enough to measure the overall physical health of dialysis patients. For instance, the dialysis patients were encouraged to engage in moderate activities to maintain the body’s functions. Instead of asking whether their health restricted the patients to performing ‘moderate activities’, the HK specific version was changed to ‘vigorous activities’. Because ’to climb several flights of stairs’ might not be applicable to most Hong Kong people, as escalators are available in most apartment buildings, ‘to walk several blocks’ was therefore included in the specific scale. Instead of asking ‘How much does pain interfere with your normal work?’, the selected item was changed to ‘How much pain have you had during the past 4 weeks?’ The revised item selection was able to measure an individual’s physical abilities to perform certain activities in daily life. Moreover, it is easily comprehensible and relevant to the living circumstances in Hong Kong. The items for the PCS in the standard SF-12 may not be an equivalent substitute for the SF-36 for Chinese dialysis patients. The items could be revised using the HK specific version to ensure its equivalence.

**Table 7:** **Comparisons of SF-36 and Chinese (HK) specific SF-12**

PCS	Mean ± SD
SF-36	27.44 ± 13.35
Chinese (HK) specific SF-12	31.69 ± 11.33
Difference	-4.25
Correlation	0.876**
Effect size	0.32
MCS	
SF-36	42.92 ± 13.86
Chinese (HK) specific SF-12	42.86 ± 12.58
Difference	-1.81
Correlation	0.901**
Effect size	0.04

Spearman’s rho correlation, **p < 0.01.

Our results supported the hypotheses on convergent and discriminant validity. A correlation coefficient of above 0.4 for convergent validity is considered satisfactory [28]. There were significant negative correlations between the disease-specific domain scores and the depression score, with the MCS having the highest correlation with depression, at *r* = -0.516. With regard to symptoms and problems, the effects of kidney disease and the burden of kidney disease, the correlations were moderate. The nature and progression of end stage renal disease causes patients to get used to the idea that they will need lifelong treatment and to accept the disruption in their daily life activities [29]. As a result, the patients were less depressed compared with the newly diagnosed patients, even though they were bothered by the symptoms and impacts of kidney disease in their daily life. A relatively low correlation was found between the PCS and depression. A possible explanation for this is that the items selected in the Standard SF-12 are not sensitive enough to capture the situation for dialysis patients. On the other hand, the evidence shows that anxiety is common in patients on maintenance dialysis and that this aspect is understudied [30]. In our study, anxiety levels were less prominent with regard to the question of whether kidney disease is a burden to patients and their families due to the prolonged trajectory of the illness. For discriminant validity, the scale is able to discriminate between the QoL of dialysis and transplant patients, producing significantly different results. The results corroborated those of previous studies confirming that transplant RRT provides a better QOL compared with other replacement methods [31].

The KDQOL-36™ is considered reliable and to have good reproducibility, as indicated by the high ICC value of >0.98 in all of the subscales. For test-retest reliability, an ICC of 0.70-0.86 demonstrated the stability of the scale over time [32]. The Kappa Index of 0.68-1 and Weighted Kappa of 0.73-1 indicated a substantial to perfect agreement across various items in test and retest reliability [33]. The Cronbach’s alpha values suggested that the scale is internally reliable. The internal reliability of all of the subscales exceeded 0.65, with the exception of the PCS and MCS. As mentioned, the item selection for the PCS may need to be revised using the Hong Kong specific version to replace the standard version. The relatively low Cronbach’s alpha for the MCS could have been affected by the fewer items in the scale. Any instrument with more than 14 items may have a higher Cronbach’s alpha value even if the items reflect different underlying constructs [34].

## Conclusions

This study showed that the Cantonese Chinese KDQOL-36 has relatively good reliability and modest validity. It is less sensitive at measuring the PCS scores for evaluating general physical health. It is recommended that the HK specific version be used to replace the Standard SF-12 for items contributing to the PCS. Testing of the reliability and validity of the scale should be an ongoing process. A larger sample size should be used, the items for the PCS should be replaced, and a standalone KDQOL-36 should be used to confirm the psychometric performance of the scale. All of the above recommendations should be carefully considered before it can be stated that the Cantonese Chinese KDQOL-36 is a responsive instrument for monitoring the QoL of chronic kidney disease patients in Hong Kong.

## References

[1] White SL, Chadban SJ, Jan S, Chapman JR, Cass A: *How can we achieve global equity in provision of renal replacement therapy?*. http://www.who.int/bulletin/volumes/86/3/07-041715/en/. (accessed 8 Aug 2014) 10.2471/BLT.07.041715 PMC2647401 18368211

[2] Ho YW, Chau KF, Choy BY, Fung KS, Cheng YL, Kwan TH, Wong PN, Lai WM, Yuen SK, Lo SHK, Chan CK, Leung CB (2013). Hong Kong Renal Registry Report 2012. Hong Kong J Nephrol.

[3] Merkus MP, Jager KJ, Dekker FW, Boeschoten EW, Stevens P, Krediet RT (1997). Quality of life in patients on chronic dialysis: self-assessment 3 months after the start of treatment. Am J of Kidney Dis.

[4] Finkelstein FO, Wuerth D, Finkelstein SH (2009). Health related quality of life and the CKD patient: challenges for the nephrology community. Kidney Int.

[5] Hays RD, Kallich JD, Mapes DL, Coons SJ, Carter WB (1994). Development of the Kidney Disease Quality of Life (KDQOL™) Instrument. Qual Life Res.

[6] Lacson E, Xu J, Lin SF, Dean SG, Lazarus JM, Hakim RM (2010). A comparison of SF-36 and SF-12 composite scores and subsequent hospitalization and mortality risks in long-term dialysis patients. Clin J Am Soc Nephrol.

[7] *Cantonese*. http://www.omniglot.com/chinese/cantonese.htm. (accessed 15 Dec 2014)

[8] *Medical Education Institute, Inc. Measuring Dialysis Patients’ Health-Related Quality of Life with the KDQOL-36™*. http://kdqol-complete.org/pdfs/kdqol-36.pdf

[9] Javali SB, Gudaganavar NV, Raj SM: **Effect of varying sample size in estimation of reliability coefficients of internal consistency.** *WebmedCentral Biostatistics* 2011.,**2**(2)**:** WMC001649

[10] Hobart JC, Cano SJ, Warner TT, Thompson AJ (2012). What sample sizes for reliability and validity studies in neurology?. J Neurol.

[11] Bannigan K, Watson R (2009). Reliability and validity in a nutshell. J Clin Nurs.

[12] Lam CL, Gandek B, Ren XS, Chan MS (1998). Test of scaling assumptions and construct validity of the Chinese (HK) version of the SF-36 Health Survey. J Clin Epidemiol.

[13] Lam CL, Tse EY, Gandek B, Fong DY (2005). The SF-36 summary scales were valid, reliable, and equivalent in a Chinese population. J Clin Epidemiol.

[14] Fong DY, Lam CL, Mak KK, Lo WS, Lai YK, Ho SY, Lam TH (2010). The Short Form-12 Health Survey was a valid instrument in Chinese adolescents. J Clin Epidemiol.

[15] Lam CLK, Wong CKH, Lam ETP, Lo YYC, Huang WW (2010). Population norm of Chinese (HK) SF-12 health survey-version 2 of Chinese adults in Hong Kong. Hong Kong Pract.

[16] Lam CL, Eileen YY, Gandek B (2005). Is the standard SF-12 health survey valid and equivalent for a Chinese population?. Qual Life Res.

[17] Hoekstra RA, Bartels M, Cath DC, Boomsma DI (2008). Factor structure, reliability and criterion validity of the Autism-Spectrum Quotient (AQ): A study in Dutch population and patient groups. J Autism Dev Disord.

[18] Schrag A (2006). Quality of life and depression in Parkinson's disease. J Neurol Sci.

[19] Finkelstein FO, Finkelstein SH (2000). Depression in chronic dialysis patients: assessment and treatment. Nephrol Dial Transpl.

[20] Boylan LS, Flint LA, Labovitz DL, Jackson SC, Starner K, Devinsky O (2004). Depression but not seizure frequency predicts quality of life in treatment-resistant epilepsy. Neurology.

[21] Abd ElHafeez S, Sallam SA, Gad ZM, Zoccali C, Torino C, Tripepi G, ElWakil HS, Awad NM (2012). Cultural adaptation and validation of the “Kidney Disease and Quality of Life-Short Form (KDQOL-SF™) version 13” questionnaire in Egypt. BMC Nephrol.

[22] Tomasz W, Piotr S (2002). A trial of objective comparison of quality of life between chronic renal failure patients treated with hemodialysis and renal transplantation. Annals of Transplantation: Quarterly of the Polish Transplantation Society.

[23] Duarte PS, Ciconelli RM, Sesso R (2005). Cultural adaptation and validation of the “Kidney Disease and Quality of Life-Short Form (KDQOL-SF™ 1.3)” in Brazil. Nraz J Med Biol Res.

[24] Lam ET, Lam CL, Fong DY, Huang WW (2013). Is the SF‒12 version 2 Health Survey a valid and equivalent substitute for the SF‒36 version 2 Health Survey for the Chinese?. J Eval Clin Pract.

[25] Porter AC, Vijil JC, Unruh M, Lora C, Lash JP (2010). Health-related quality of life in Hispanics with chronic kidney disease. Transl Res.

[26] Lam CLK, Wong CKH, Lam ETP, Lo YYC, Huang WW (2010). Population norm of Chinese (HK) SF-12 health survey-version 2 of Chinese adults in Hong Kong. The Hong Kong Practitioner.

[27] Norman GR, Sridhar FG, Guyatt GH, Walter SD (2001). Relation of distribution-and anchor-based approaches in interpretation of changes in health-related quality of life. Med Care.

[28] Kaasa S, Bjordal K, Aaronson N, Moum T, Wist E, Hagen S, Kvikstad A (1995). The EORTC core quality of life questionnaire (QLQ-C30): validity and reliability when analysed with patients treated with palliative radiotherapy. Eur J Cancer.

[29] Polaschek N (2003). The experience of living on dialysis: a literature review. Nephrology Nurs J.

[30] Feroze U, Martin D, Reina-Patton A, Kalantar-Zadeh K, Kopple JD (2010). Mental health, depression, and anxiety in patients on maintenance dialysis. Iran J Kidney Dis.

[31] Ogutmen B, Yildirim A, Sever MS, Bozfakioglu S, Ataman R, Erek E, Cetin O, Emel A (2006). Health-related quality of life after kidney transplantation in comparison intermittent hemodialysis, peritoneal dialysis, and normal controls. Transplant Proc.

[32] Portney LG, Watkins MP (2009). Foundations of Clinical Research: Applications to Practice.

[33] Viera AJ, Garrett JM (2005). Understanding interobserver agreement: The kappa statistic. Fam Med.

[34] Cortina JM (1993). What is coefficient alpha? An examination of theory and applications. J Appl Psychol.

[CR35] The pre-publication history for this paper can be accessed here: http://www.biomedcentral.com/1471-2369/15/199/prepub

